# Sialidase NEU3 Contributes to the Invasiveness of Bladder Cancer

**DOI:** 10.3390/biomedicines12010192

**Published:** 2024-01-16

**Authors:** Takeo Tatsuta, Jun Ito, Koji Yamamoto, Shigeki Sugawara, Masahiro Hosono, Makoto Sato, Taeko Miyagi

**Affiliations:** 1 Division of Cell Recognition Study, Tohoku Medical and Pharmaceutical University, Sendai 981-8558, Japan; t-takeo@tohoku-mpu.ac.jp (T.T.); ssuga@tohoku-mpu.ac.jp (S.S.); mhosono@tohoku-mpu.ac.jp (M.H.); 2 Department of Urology, Faculty of Medicine, Tohoku Medical and Pharmaceutical University, Sendai 983-8536, Japan; itojun@tohoku-mpu.ac.jp (J.I.); ms.hifu@tohoku-mpu.ac.jp (M.S.); 3 Faculty of Health and Medical Care, Saitama Medical University, Saitama 350-0496, Japan; yama_ko@saitama-med.ac.jp; 4 Division of Cancer Chemotherapy, Miyagi Cancer Center Research Institute, Natori 981-1293, Japan

**Keywords:** sialidase, invasion, bladder cancer

## Abstract

Bladder cancer is the 10th most commonly diagnosed cancer worldwide. The current standard treatment for advanced bladder cancer is neoadjuvant cisplatin (NAC)-based chemotherapy followed by cystectomy. However, the response rate to chemotherapy is only 50%, owing to cisplatin resistance, and there is a need for novel therapies. Because the invasiveness of bladder cancer greatly influences patient prognosis, a mechanistic analysis of the invasive function can lead to therapeutic targets. Sialidases, which remove sialic acid residues from the nonreducing ends of sugar chains and catalyze the initial reaction in the degradation of sugar chains, are predicted to be involved in cell invasion and motility. However, the involvement of sialidases in bladder cancer, especially their relationship with the invasive ability, remains unclear. Here, using patient tissues and multiple bladder cancer cell lines, we show that the sialidase *NEU3* is highly expressed in bladder cancer. Analysis of NEU3’s function using its siRNA-mediated knockdown revealed that NEU3 contributes to bladder cancer invasiveness. Mechanistic analysis showed that NEU3 activates ERK and PI3K signaling. Our results show that NEU3 is involved in the malignancy of bladder cancer, and its suppression may lead to novel treatments for bladder cancer.

## 1. Introduction

Bladder cancer is the 10th most common cancer worldwide [1]. Urothelial carcinoma (also called transitional cell carcinoma) is the most common type of bladder cancer, accounting for more than 90% of the cases, and is classified as non-muscle-invasive bladder cancer or muscle-invasive bladder cancer, depending on the nature of the tumor [2]. Non-muscle-invasive bladder cancer has a high recurrence rate, and in approximately 10–15% of the cases, the cancer progresses to the muscle-invasive type, which is characterized as a metastatic malignant tumor. Once muscle-invasive bladder cancer progresses to metastatic bladder cancer, the 5-year survival rate decreases to 5% [3]. Cisplatin-based combination chemotherapy has been widely used to treat muscle-invasive bladder cancer, and it increases survival rates [4]. However, the response rate to chemotherapy is only 50%, and cisplatin resistance is believed to be the cause of this low response rate [5].

Cancer cell migration and invasion are dynamic and complex processes that contribute to the progression of many tumors. The ability of cancer cells to migrate and invade allows them to relocate within tissues and detach from the primary tumor, resulting in the spread of the disease. The invasiveness of bladder cancer is a significant factor in its malignancy that dramatically reduces the survival rate. Therefore, there is a need to develop new therapeutic agents that suppress the invasion and metastasis of bladder cancer, and to elucidate the underlying molecular mechanisms. 

Sialidases are glycosidases that remove sialic acid residues from the nonreducing ends of sugar chains and catalyze the initial reaction in the degradation of sugar chains. The removal of sialic acid by sialidases not only promotes the catabolic degradation of sugar chain molecules, but also significantly affects the conformation of sugar chain molecules, recognition by receptors, cell adhesion, and the immune system. Four types of sialidases (NEU1, NEU2, NEU3, and NEU4) have been identified in animal cells; however, their chromosomal location and enzymatic properties, such as intracellular localization and substrate specificity, are different [6]. Of the four sialidases, *NEU1* is the gene responsible for sialidosis, a sialidase-deficient disease, which encodes a sialidase present in lysosomes. NEU3 specifically hydrolyses gangliosides and is localized in the plasma membrane, having a close association with caveolin in the membrane microdomain. NEU3 is upregulated in various cancer types and is predicted to be involved in cell invasion and motility. This abnormality can lead to the impairment of cell functions. It is indeed involved in promoting cell invasion and motility in renal cell carcinoma, head and neck squamous cell carcinoma, and glioblastoma [6]. However, there are few reports on the involvement of sialidases in bladder cancer, especially regarding their relationship with the invasive ability. 

In the present study, we investigated the expression of all four sialidases in the tissues of patients with bladder cancer. The results indicated that the expression of *NEU3* is increased in bladder cancer tissues. In addition, experiments using several bladder cancer cells and normal mucosa-derived cells showed that *NEU3* is highly expressed in cancer cells, and that knockdown of *NEU3* inhibits the invasive ability of invasive bladder cancer cells. We also investigated the molecular mechanisms underlying the role of NEU3 in the invasive ability of bladder cancer cells.

## 2. Materials and Methods

### 2.1. Collection of Bladder Cancer Tissue

Tissue samples were obtained from patients with bladder cancer who were admitted to Tohoku Medical and Pharmaceutical University Hospital. Tumor (*N* = 26) and non-tumor (*N* = 12) tissue samples were collected from patients during the removal of cancer tissue via transurethral resection and stored at −80 °C until analysis. Sample collection protocols were approved by the Institutional Review Board of Tohoku Medical and Pharmaceutical University Hospital (approval code: 2022-0-013). This study was conducted in accordance with the Declaration of Helsinki and the ethical standards that promote and ensure respect and integrity for all human subjects.

### 2.2. Cell Culture

The cell lines derived from normal bladder epithelial cells (HCV29), invasive bladder carcinoma (T24), and MGH-U1 cells were purchased from the American Type Culture Collection (Rockland, MD, USA). KK-47 cells (derived from non-invasive bladder carcinoma) were donated by the Department of Pharmaceutical Sciences, Tohoku University. YTS-1 and YTS-3 cells (derived from invasive bladder carcinoma) were donated by the Department of Urology, Yamagata University School of Medicine. The cell lines were cultured in RPMI medium, supplemented with 10% heat-inactivated fetal bovine serum and antibiotic-antimycotic solution (penicillin (100 IU/mL), streptomycin (100 µg/mL), and amphotericin B (0.25 µg/mL); Life Technologies, Carlsbad, CA, USA), at 37 °C in an atmosphere of 95% air and 5% CO_2_. For silencing, siRNAs targeting NEU3 were synthesized by Dharmacon Inc. (Thermo Fisher Scientific, Waltham, MA, USA), as described previously [7], and transfected using the Lipofectamine RNAiMAX reagent (Invitrogen, San Diego, CA, USA).

### 2.3. Reverse Transcription-Quantitative Real-Time Polymerase Chain Reaction (RT-qPCR)

Total RNA was extracted from the tissues or cells using a Direct-Zol RNA MiniPrep Kit (Zymo Research, Irvine, CA, USA). cDNA was synthesized from total RNA (1 µg) using a SuperScript VILO cDNA Synthesis Kit (Invitrogen). qPCRs were performed on a LightCycler 480 system using the LightCycler 480 Probes Master Kit (Roche Diagnostics, Indianapolis, IN, USA) with specific primers for each sialidase.

### 2.4. Sialidase Activity Assays

Crude extracts were used for sialidase assays using bovine brain ganglioside GM3 (Alexis Biochemicals, Lausen, Switzerland) as a substrate in the presence of 0.1% Triton X-100. After incubation of the reaction mixture at 37 °C for 30 min, the amount of sialic acid released was determined using a modified thiobarbituric acid method, or fluorometric high-performance liquid chromatography with malononitrile [8]. One unit of activity was defined as the amount of enzyme required to cleave 1 nmol of sialic acid from the substrate. The protein concentrations were determined using a dye-binding assay (Bio-Rad Laboratories, Hercules, CA, USA).

### 2.5. Invasion Assay

Invasion assays were performed using BD Biocoat Matrigel invasion chambers (BD Biosciences, Franklin Lakes, NJ, USA). The cells were seeded at a density of 2 × 10^4^ cells/well in serum-free medium in the upper chamber, and the lower chamber was filled with medium containing 10% fetal bovine serum. After 24 h, the cells were fixed and stained with 0.1% crystal violet, and invading or migrating cells on the lower membrane surface were counted under a microscope. Data are expressed as the invasion ratio through the Matrigel matrix and membrane relative to migration through the control membrane.

### 2.6. Antibody Arrays of Phospho-RTK

An assay for the phospho-RTK array was performed using RayBio Human RTK Phosphorylation Array C1 (RayBiotech, Peachtree Corners, GA, USA) according to the manufacturer’s instructions. The reagents provided in the kit were used for the assay. Briefly, phospho-RTK array membranes were blocked with a blocking buffer and incubated with cell lysates after normalizing for protein amounts. The membranes were then incubated with biotinylated anti-phospho tyrosine detection antibody, and subsequently with streptavidin-conjugated horseradish peroxidase. Finally, each array membrane was exposed to X-ray film and the signals were visualized using a chemiluminescence detection system. The relative density of the protein bands was measured using ImageJ 1.51s software (NIH, Bethesda, MD, USA).

### 2.7. Western Blotting

Cells (5 × 10^4^ cells/mL) were cultured in 6-well plates and whole cell lysates were prepared using an extraction buffer [150 mM NaCl, 10 mM Tris-HCl (pH 7.4), 5 mM EDTA, 1% Nonidet P-40, 0.1% sodium deoxycholate, and 0.1% sodium dodecyl sulfate] supplemented with cOmplete™ Mini EDTA-free protease inhibitor cocktail tablets (one tablet/10 mL; Roche Applied Science, Madison, WI, USA). Soluble proteins were collected, and the protein concentration was measured using a BCA protein assay kit (Thermo Fisher Scientific) according to the manufacturer’s instructions. Proteins were separated using electrophoresis on 10 or 14% sodium dodecyl sulfate-polyacrylamide gels and transferred onto Immobilon-P transfer membranes (Thermo Fisher Scientific). The membranes were sequentially incubated with primary and secondary antibodies diluted in Can Get Signal solution (Toyobo, Osaka, Japan). Protein bands were detected using ImmunoStar LD Western Blotting Detection Reagent (Wako, Osaka, Japan). The antibodies against phospho-ERK, ERK, phospho-AKT, AKT, phospho-PI3K, PI3K, androgen receptor, beta-actin, and rabbit IgG used in this study were purchased from Cell Signaling Technology (Danvers, MA, USA).

### 2.8. Statistical Analysis

Results from three or more independent experiments are expressed as mean ± standard deviation. Statistical analyses were conducted using GraphPad Prism 5.0 (GraphPad Software, Inc., San Diego, CA, USA), and comparisons were made using a two-tailed Student’s *t*-test or one-way analysis of variance, followed by Bonferroni’s post hoc test. A *p*-value < 0.05 was considered to indicate a statistically significant difference.

## 3. Results

### 3.1. NEU3 Is Highly Expressed in Cancer Tissues

**Figure 1:**
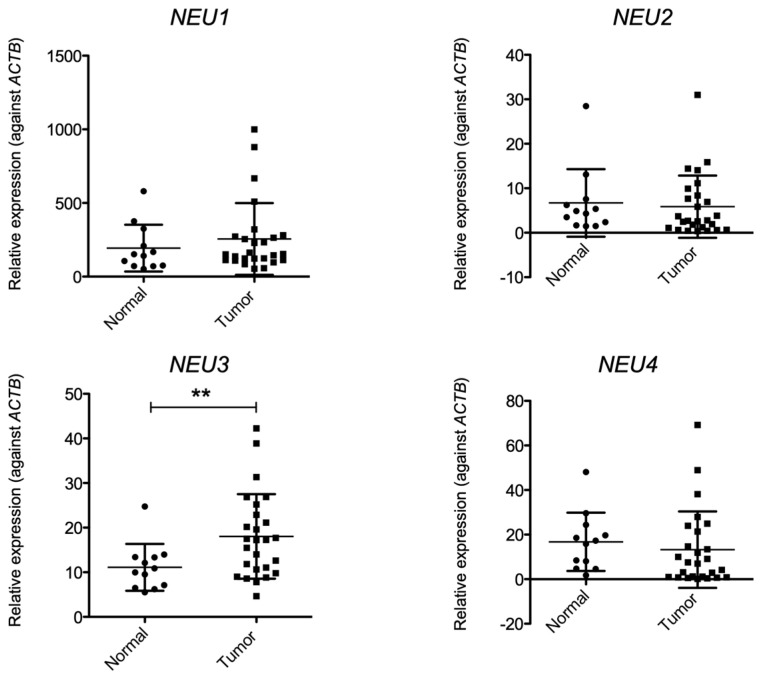
Expression of sialidases in the bladder tissue. RNA was extracted from normal (*N* = 12) or tumorous (*N* = 26) bladder tissue. Quantitative RT-PCR was performed using specific primers to evaluate the expression of sialidases. The expression levels were normalized against ACTB. Data given are mean ± S.D values from three independent experiments performed in triplicate. ** *p* < 0.005.

**Figure 2:**
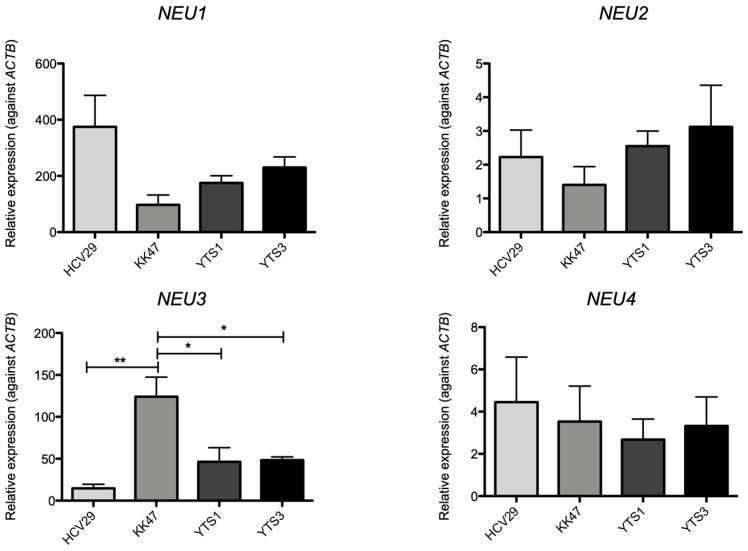
Expression of sialidases in bladder cells. Quantitative RT-PCR was performed using specific primers to evaluate the expression of sialidases. The expression levels were normalized against ACTB. Data given are mean ± S.D values from three independent experiments performed in triplicate. * *p* < 0.05, ** *p* < 0.005.

**Figure 3:**
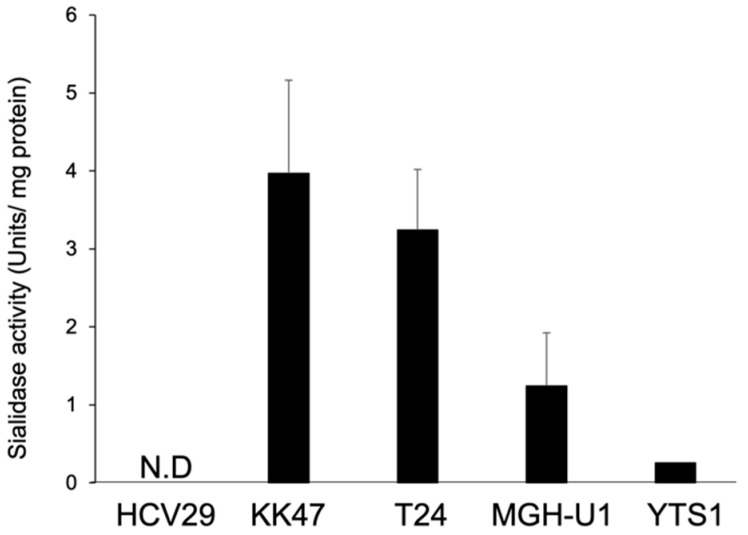
Sialidase activity in bladder cells. NEU3 activity was determined using GM3 as a substrate. Data given are mean values of three independent experiments. N.D.; not detected.

First, we investigated sialidase expression in tissues collected from patients with bladder cancer using real-time PCR. No significant differences were observed in the expression levels of *NEU1*, *NEU2*, and *NEU4* in any of the bladder cancer tissues tested in this study compared with those in normal cells. In contrast, the expression level of *NEU3* was significantly higher in tumor tissues than in normal tissues (Figure 1). Next, we determined the expression of sialidase in the different selected cell lines. The expression of the four types of sialidases in each cancer cell line (KK47, YTS1, and YTS3) was measured and compared with that in normal cells (HCV29). The expression of *NEU1* tended to decrease in cancer cells, and it was particularly low in KK47 cells. No major differences in the expression of *NEU2* and *NEU4* were observed among the cell lines. The expression of *NEU3* was significantly higher in KK47 than in other cell lines. Although there was no significant difference, the expression of *NEU3* in YTS1 and YTS3 was higher than that in HCV29 (Figure 2). We also investigated endogenous NEU3 activity in each cell line (Figure 3). In addition to HCV29, KK47, and YTS1, cell lines which exhibit invasive properties—T24 and MGHU1—were also used. The NEU3 activity showed a trend similar to that of mRNA expression. The activity in different preparations of HCV29, a noncancerous cell line, was measured three times; however, it remained below the detection limit. In contrast, NEU3 activity was confirmed in all of the four cancer cell lines. These results indicate that the expression and activity of NEU3 are enhanced in bladder cancer.

### 3.2. Reduced Expression of NEU3 Suppresses Invasion of Invasive Bladder Cancer Cells

**Figure 4:**
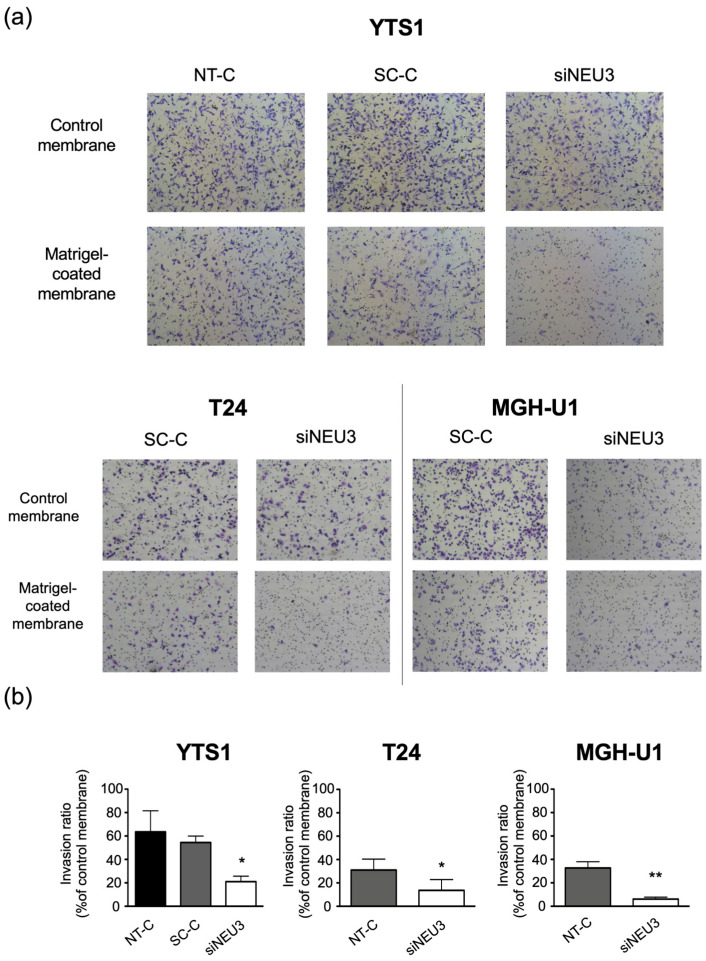
Suppression of invasiveness through siRNA-mediated silencing of *NEU3* in YTS1, T24, and MGH-U1 cells. The cells were transfected with siRNAs targeting *NEU3* (siNEU3), nontarget siRNA control (NT-C), or scrambled control (SC-C) for 24 h, and the invasion assay was performed. (**a**) Representative images of invasion assay using control or Matrigel-coated Transwell. (**b**) The invasion ratio was determined for each cell line. Each bar represents the mean ± S.D. of values from three experiments. * *p* < 0.05, ** *p* < 0.01.

Because *NEU3* was found to be highly expressed in bladder cancer, we investigated whether it was involved in the invasive ability of bladder cancer. At the preliminary testing stage, no invasive properties of KK47 cells were observed using the chamber assay system, consistent with the nature of the cells’ origin (Figure S1). Therefore, the invasive ability of YTS1, T24, and MGH-U1 cells knocked down for *NEU3* was measured. First, to confirm the knockdown efficiency of siNEU3, the expression level of NEU3 in YTS1 cells was confirmed by qRT-PCR under the same conditions as in the invasion assay. siRNA-transfected cells were harvested 24 h later, then the cells were replated and cultured for an additional 24 h. After that, RNA was extracted and the expression level of NEU3 was quantified. Compared to control cells (NT-C and SC-C), the expression level of NEU3 was approximately 20%, which was a significant decrease (Figure S2a). Furthermore, when measuring enzymatic activity, siNEU3 treatment reduced NEU3 activity in YTS1 cells to below the detection limit (Figure S2b). Therefore, we conducted experiments under these conditions. *NEU3* knockdown and control YTS1, T24, and MGH-U1 cells showed migration and invasion (Figure 4a). After the invasion assay, cells that had invaded the bottom of the chamber were observed, the number of cells was counted, and the percentage (%) invasion ability was calculated (Figure 4b). The % invasion ability of YTS-1 cells was 63.5% for the nontarget control (NT-C), 54.5% for the scrambled control (SC-C), and 21.1% for the siNEU3-treated cells. Similarly, the % invasion ability was 31.1% for SC-C- and 13.6% for siNEU3-treated T24 cells, and 32.7% and 6.1% for SC-C- and siNEU3-treated MGH-U1 cells, respectively. These results confirmed that the invasive ability of all invasive bladder cancer cells used in this study was significantly reduced upon siNEU3 treatment compared with that of control cells.

### 3.3. Knockdown of NEU3 Induces a Decrease in Invasive Ability and Is Accompanied by the Suppression of ERK and PI3K Activation

**Figure 5:**
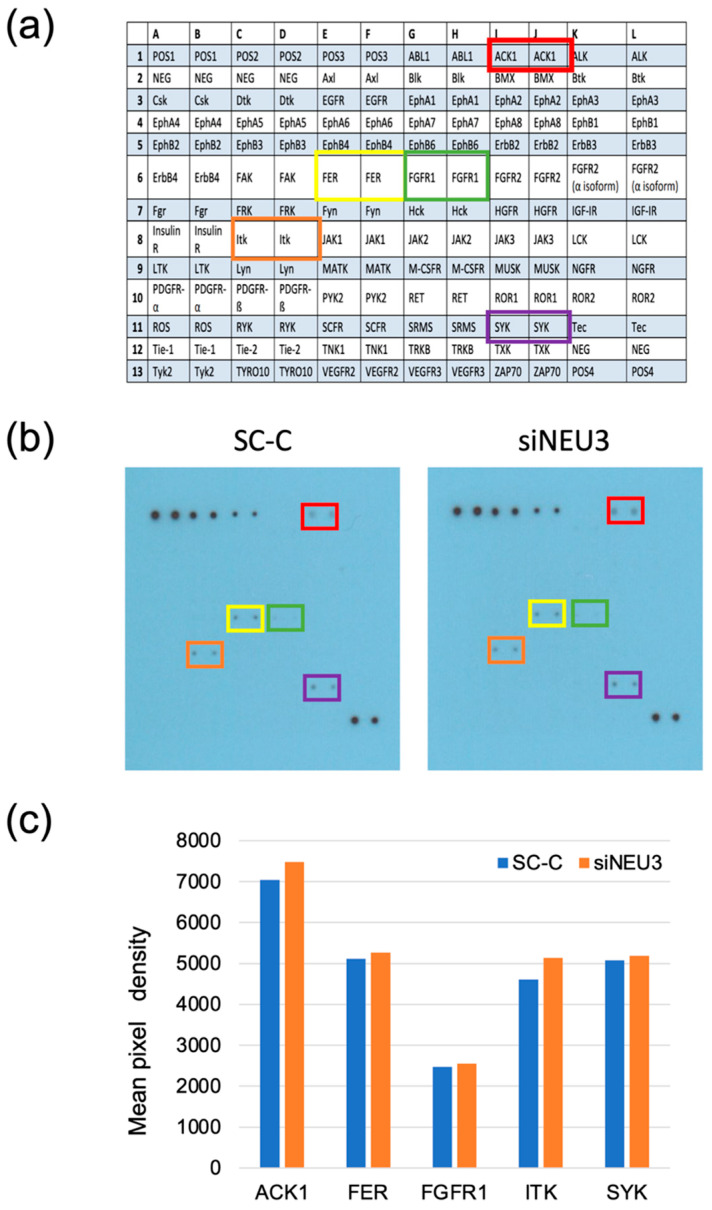
Comprehensive analysis of the phosphorylation status of tyrosine kinases (TKs) in YTS-1 cells. The cells were transfected with siRNAs targeting *NEU3* (siNEU3) or scrambled control (SC-C) for 24 h. Whole cell lysate was extracted and array analysis was performed. (**a**) List of TKs on the array membrane. The phosphorylation of TKs that were detected is highlighted in color. (**b**) Image of the membranes showing phosphorylation. (**c**) Histograms for the quantification of signals in the antibody array.

**Figure 6:**
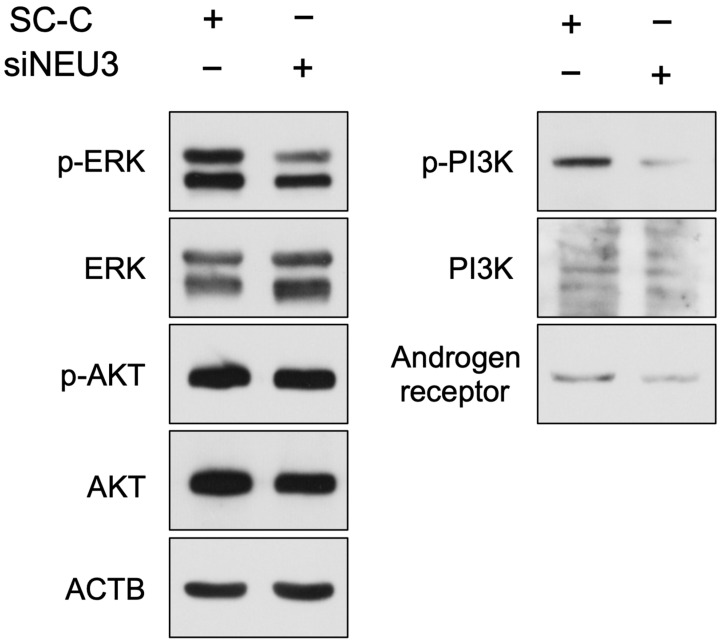
Effect of siNEU3 on the invasion-related molecules in YTS-1 cells. The cells were transfected with siRNAs targeting *NEU3* (siNEU3) or scrambled control (SC-C) for 24 h, and the expression levels of p-ERK, ERK, p-AKT, AKT, p-PI3K, PI3K, and androgen receptor were detected using western blotting. ACTB was used as an internal control.

Knockdown of *NEU3* reduced the invasive ability of bladder cancer cells. Therefore, we investigated the molecular mechanisms underlying the decrease in the invasive ability. Several receptor tyrosine kinases (RTKs), such as fibroblast growth factor receptor (FGFR) 1 and 3 [8], human epidermal growth factor receptor 2 (HER2) [9], insulin-like growth factor-1 receptor (IGF1R) [9], and ephrin type-B receptor 4 (EphB4) [9], have been reported to be involved in the invasion of bladder cancer. To verify whether the activation of RTKs is related to the decreased invasive ability of invasive bladder cancer cells, we used the RayBio Human Receptor Tyrosine Kinases (RTKs) Phosphorylation Antibody Array to investigate the activation of RTKs in siNEU3-treated cells, and the phosphorylation status 71 receptor of non-RTKs were comprehensively analyzed. Activated Cdc42-associated kinase 1 (ACK1), proto-oncogene tyrosine-protein kinase (FER), FGFR1, interleukin-2-inducible T-cell kinase (ITK), and spleen tyrosine kinase (SYK) were phosphorylated in YTS-1 cells under our culture conditions; however, no differences were observed between control cells and siNEU3-treated cells (Figure 5). Therefore, NEU3 does not affect the activation status of these TKs. Activation of ERK and PI3K by various stimuli has also been reported to be involved in the invasion of bladder cancer. Therefore, we investigated these signaling molecules in siNEU3-treated cells using western blotting (Figure 6). These results indicate that the phosphorylation of ERK and PI3K was reduced upon siNEU3 treatment, suggesting that NEU3 may be involved upstream of their activation. Although there was no major change in AKT phosphorylation, a slight decrease in total AKT levels was observed. We also examined the expression of the androgen receptor, which has been reported to be involved in bladder cancer invasion [10], and found that it was decreased in siNEU3-treated cells. These results indicate that decreased NEU3 expression causes a decline in invasive ability, and is accompanied by suppression of ERK and PI3K activation. Although NEU3 does not affect the activity of TKs, it is possibly related to downstream signals of RTKs and is involved in the expression of the androgen receptor.

## 4. Discussion

We demonstrate for the first time that *NEU3* is highly expressed in bladder cancer. In multiple invasive bladder cancer cells, we found that knockdown of *NEU3* reduced the invasive ability. Thus, NEU3 is involved in the invasiveness of bladder cancer. *NEU3* knockdown did not affect the phosphorylation of TKs in YTS-1 cells. In contrast, siNEU3 treatment reduced the activation or expression of ERK, PI3K, and androgen receptor, which are involved in the invasiveness of bladder cancer. Therefore, it is possible that NEU3 is highly expressed in bladder cancer and controls the activity of the abovementioned signaling molecules without affecting TKs.

The presence or absence of invasive ability in bladder cancer is one of the major factors determining prognosis, and is also the key to selecting a cancer treatment strategy. While treatments that preserve the bladder’s function—such as transurethral resection of bladder tumor—are generally used for superficial bladder cancer, for invasive bladder cancer, radical cystectomy is used to remove cancer cells to the furthest extent possible. As the survival rate of patients who undergo total resection is low, multimodal treatment centered on surgery is used for invasive bladder cancer. However, the main problem with superficial bladder cancer is its tendency to recur; some patients may undergo pathological changes during repeated recurrences, resulting in invasive cancer [11]. Therefore, molecules that suppress the acquisition of the invasive ability by cancer cells and those that contribute to the acquired invasive ability are considered important therapeutic targets for bladder cancer. In this study, all the selected cancer cell lines showed higher expression or activity of NEU3 than that in normal tissue-derived cell lines, although there were differences depending on the cell line (Figure 2). As mentioned above, NEU3 may affect the invasiveness of bladder cancer cells; however, KK47, a superficial bladder cancer cell line, showed high expression and activity of NEU3. Although the reason for this is unclear, NEU3 may have functions other than invasiveness, such as contributing to the carcinogenicity of KK47 cells. Indeed, NEU3 has been reported to be involved in carcinogenesis, as evident from the fact that NEU3 transgenic mice have increased azoxymethane-induced abnormal crypt foci, whereas NEU3 knockout mice show reduced tumor incidence in azoxymethane- and dextran sodium sulfate-induced colitis-associated colon carcinogenesis models [12].

In this study, we focused on the invasiveness of bladder cancer and investigated the involvement of NEU3. Interestingly, Watanabe et al. reported that overexpression of GM3 reduces the invasive potential of murine bladder cancer cells transfected with GM3 synthase [13], as GM3 is a good substrate of NEU3. Various studies have been conducted on the invasiveness of bladder cancer, and many reports have pointed out the involvement of the Ras/MAPK and PI3K/AKT signaling pathways [14,15,16]. In addition to the references cited in the results section, Li et al. found that aldolase A, an enzyme involved in glycolysis, regulates the invasion of bladder cancer cells via E-cadherin-EGFR signaling [17]. They showed that knockdown of aldolase A reduced the invasion of invasive bladder cell lines T24 and RT4 by decreasing EGFR, ERK, and AKT serine/threonine kinase (AKT) phosphorylation levels. Furthermore, Biswas et al. reported that knockdown of tweety family member 3 (TTYH3), a calcium-activated chloride channel, led to the downregulation of Ras/ERK signaling by inhibiting FGFR1 phosphorylation, resulting in decreased invasiveness. In our experiments, *NEU3* knockdown similarly attenuated the activation of ERK and PI3K. However, EGFR phosphorylation was not detected in YTS-1 cells, and *NEU3* knockdown had no effect on the level of phosphorylation of FGFR1, where phosphorylation was detected (Figure 5). This suggests that the invasiveness-related functions of NEU3 are operative upstream of the Ras/MAPK and PI3K/AKT signals. Moreover, in recent years, it has been reported that androgen receptor activation is positively involved in the invasiveness of bladder cancer; androgen receptor signals promote the motility and invasiveness of bladder cancer cells in various cells, and knockdown of androgen receptor has been shown to reduce motility and invasion ability [10]. Although there are still many unknowns about the mechanism, in this study, knockdown of NEU3 caused a decrease in invasion ability accompanied by a decrease in androgen receptor expression, suggesting that NEU3 may play some role upstream of this androgen receptor signal. We found that NEU3 may be a target that controls the invasive ability of bladder cancer, but due to the limited number of patient samples, we were unable to directly prove this in cancer tissue. A direct way to prove this is to investigate the activity of NEU3 in patient tissues and conduct experiments on the cells cultured from patients with the signals we have uncovered here.

NEU3 is a plasma membrane-associated ganglioside-specific sialidase. Its aberrant expression is related to the pathogenesis of cancer, as it plays a role in controlling transmembrane signaling by modulating gangliosides [6]. We previously demonstrated that NEU3, EGFR, and Src were co-immunoprecipitated in NEU3- and EGFR-transfected NIH-3T3 cells, and the overexpression of NEU3 could cause the constitutive activation of EGFR together with Src activation in the presence of EGF, leading to potentiation of the tumorigenicity of cancer cells [18]. In contrast to various carcinomas, we also reported a case in which NEU3 expression was reduced in cancer cells. NEU3 is downregulated in human glioblastoma cells, and siRNA-mediated *NEU3* silencing in glioblastoma cells enhances their invasiveness compared to control cells. Mechanistic analysis of the increase in cell invasion due to the downregulation of sialidase in these cells revealed that it involved accelerated degradation of focal adhesion proteins following calpain activation, accompanied by the accumulation of GM3. There are differences in the types and amounts of growth factor receptors and gangliosides expressed depending on the cell type, which may cause differences in the role assayed by NEU3. In this study, we found that ACK1, FER, FGFR1, ITK, and SYK were phosphorylated in YTS-1 cells, whereas NEU3 expression did not affect their behavior. TERT gene promoter, FGFR3 mutations, and HER2 and HER3 mutations were recently identified as driver genetic abnormalities in bladder cancers [19,20]. It is necessary to conduct studies using multiple cell lines to compare the expression status of growth factor receptors and gangliosides with NEU3’s function. In particular, in recent years, there have been many reports that abnormally increased FGFR3 expression is associated with bladder cancer and is attracting attention as a new therapeutic target [21]. Because the TK array used in this study did not contain FGFR3, the relationship between FGFR3 and NEU3 remains unknown and is a limitation of this study; it is possible that NEU3 is involved in FGFR3 signaling. Our results indicate that NEU3 positively functions in the malignancy of cancer, at least upstream of Ras/MAPK and PI3K/AKT, and we believe that it represents a new avenue for the treatment of bladder cancer.

## 5. Conclusions

*NEU3* is highly expressed in bladder cancer tissues compared with normal tissues and promotes the invasive ability of invasive bladder cancers. The inhibition of NEU3 can suppress the invasion of invasive bladder cancer, and might be a novel therapeutic target for refractory bladder cancer.

## Data Availability

The datasets generated in the current study are available from the corresponding author upon reasonable request.

## Supplementary Materials

The following supporting information can be downloaded at: https://www.mdpi.com/article/10.3390/biomedicines12010192/s1, Figure S1: KK47 and YTS1 cell invasion assay with Transwell chambers; Figure S2: The levels of NEU3 mRNA after siRNA transfection.
