# Supplementary material for: Sialidase NEU3 Contributes to the Invasiveness of Bladder Cancer
Source: Biomedicines. 2024 Jan 16;12(1):192. doi: 10.3390/biomedicines12010192 (PMC10813053; doi:10.3390/biomedicines12010192)
Supplement: Supplementary file 1 [file biomedicines-12-00192-s001.zip › Supplementary figures.pdf]

Supplementary figures

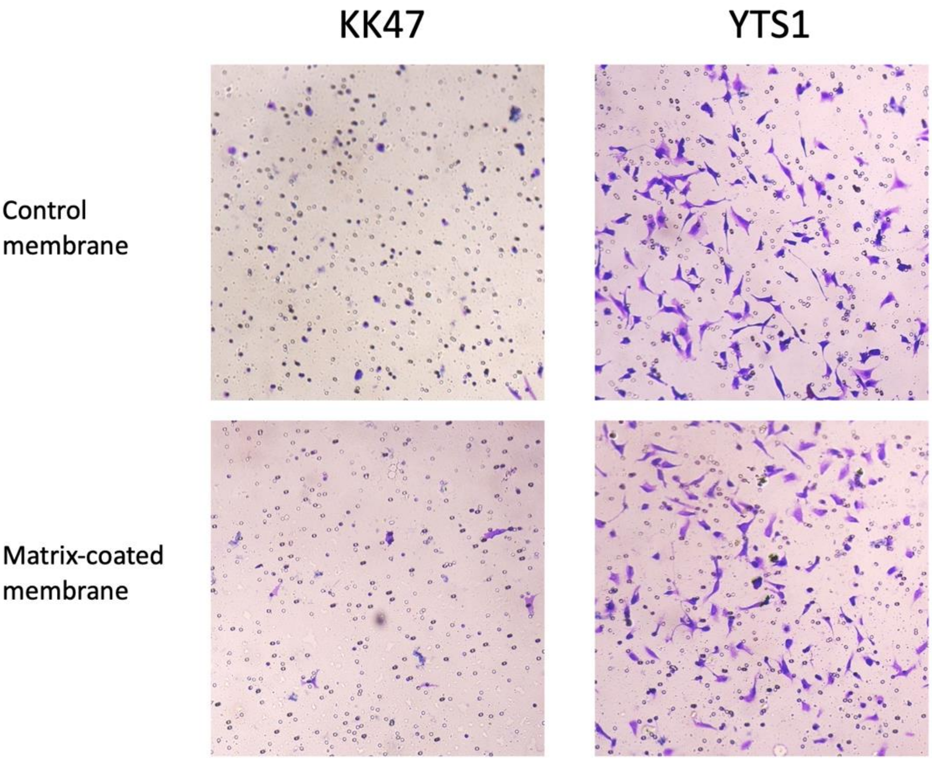

Figure S1. KK47 and YTS1 cell invasion assay with transwell chambers. Cells were seeded in control or matrigel-coated transwell (8 μm pores) at 2.5 × 10^4/well with serum-free medium on to the upper chamber, and lower chamber was filled with medium containing 10% fetal bovine serum. After 24 h, cells were fixed and stained with 0.1 % crystal violet and the migrating or invading cells on the lower membrane surface were photographed.

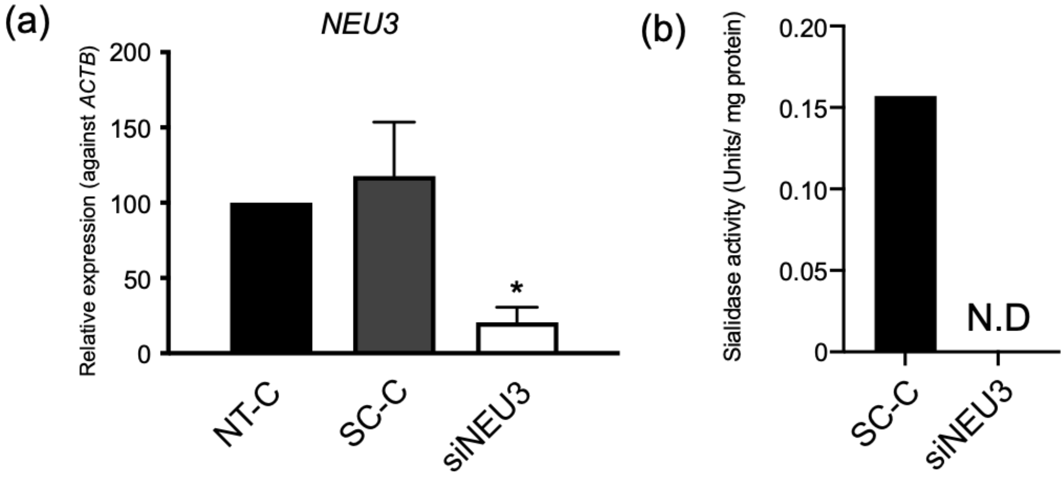

Figure S2. The levels of NEU3 mRNA and enzymatic acitivity after siRNA transfection. (a) YTS1 cells were transfected with siRNAs targeting NEU3 (siNUE3), non-targeting siRNA control (NT-C) and scrambled control (SC-C). After 24 hr transfection, cells were harvested and 1×10^5 cells were seeded in 6 well plates. After 24 hr incubation, the expressions of NEU3 were determined by qRT-PCR. NEU3 expressions were normalized by ACTB expression. Each bar represents the mean ± S.D. of three experiments. * $p < 0.05$. (b) NEU3 activity in SC-C or siNEU3 treated YTS1 cells was aldo determined using GM3 as a substrate. N.D.; not detected.
